# Correction: To isolate or not to isolate: the impact of changing behavior on COVID-19 transmission

**DOI:** 10.1186/s12889-022-14406-z

**Published:** 2022-11-11

**Authors:** Folashade B. Agusto, Igor V. Erovenko, Alexander Fulk, Qays Abu-Saymeh, Daniel Romero-Alvarez, Joan Ponce, Suzanne Sindi, Omayra Ortega, Jarron M. Saint Onge, A. Townsend Peterson

**Affiliations:** 1 University of Kansas, Lawrence, KS 66045 USA; 2 University of Kansas Medical Center, Kansas City, KS 66160 USA; 3 Purdue University, West Lafayette, IN 47907 USA; 4 University of California Merced, Merced, CA 95343 USA; 5 Sonoma State University, Rohnert Park, CA 94928 USA; 6 University of North Carolina at Greensboro, Greensboro, NC 27412 USA

**Correction: BMC Public Health 22, 138 (2022)**

**https://doi.org/10.1186/s12889-021-12275-6**

The original publication of this article [1] contained an error in Fig. 1. The parameters from H to R were labelled wrongly.

The correct and incorrect version of this figure are available in this correction article as Figs. 1 and 2. The original article has been updated.

**Fig. 1:**
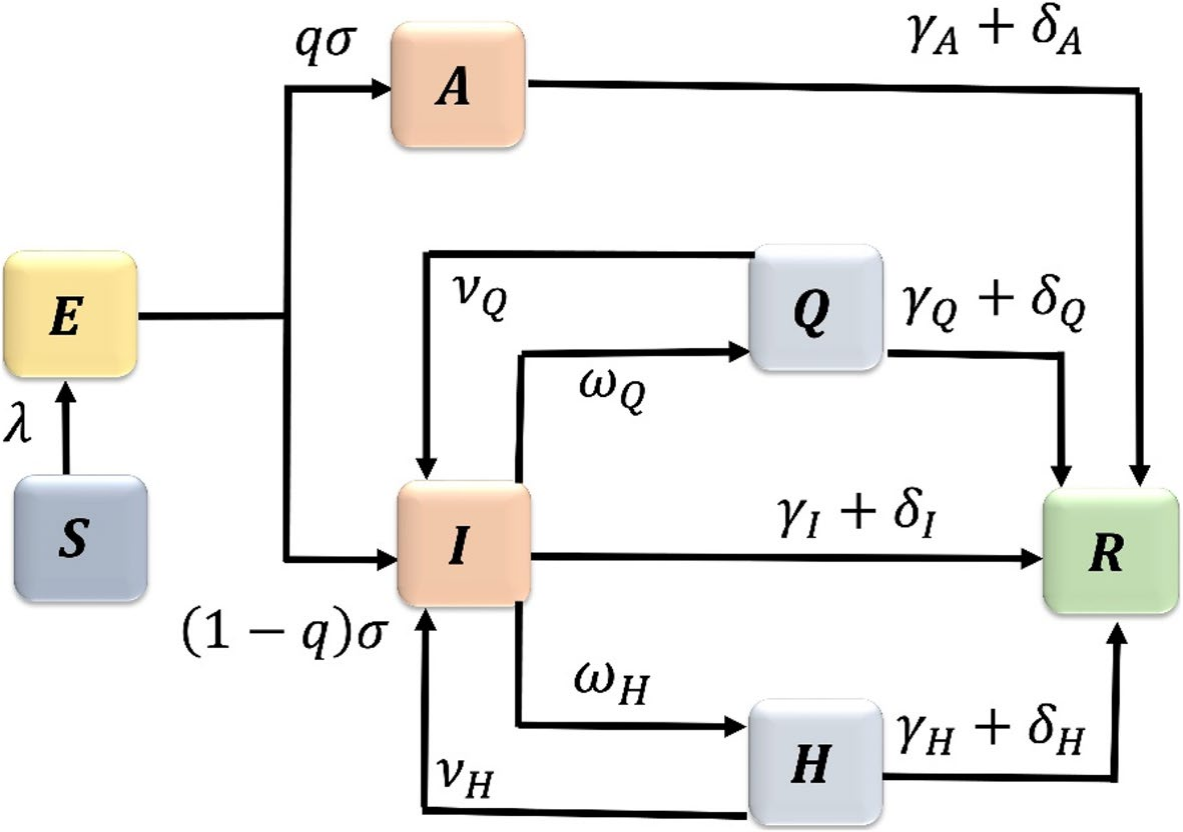
Correct version of figure 1

**Fig. 2:**
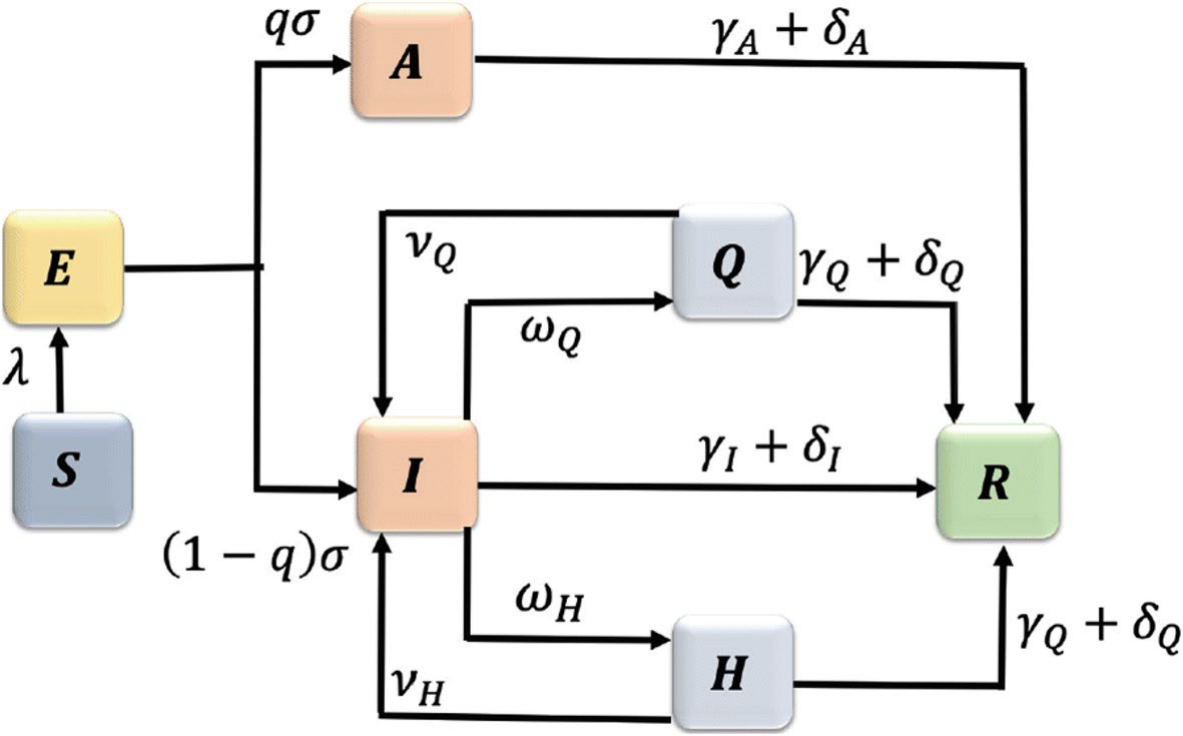
Incorrect version of figure 1 as originally published
